# State of Fluoroless Procedures in Cardiac Electrophysiology Practice

**DOI:** 10.19102/icrm.2020.110305

**Published:** 2020-03-15

**Authors:** Ugur Canpolat, Michela Faggioni, Domenico G. Della Rocca, Qiong Chen, Huseyin Ayhan, Andrew A. Vu, Sanghamitra Mohanty, Chintan Trivedi, Carola Gianni, Mohammed Bassiouny, Amin Al-Ahmad, J. David Burkhardt, Javier E. Sanchez, G. Joseph Gallinghouse, Andrea Natale, Rodney P. Horton

**Affiliations:** ^1^Texas Cardiac Arrhythmia Institute, St. David’s Medical Center, Austin, TX, USA; ^2^Arrhythmia and Electrophysiology Unit, Department of Cardiology, Hacettepe University, Ankara, Turkey; ^3^James J. Peters Veterans Affairs Medical Center, Bronx, NY, USA; ^4^Henan Provincial People’s Hospital, Zhengzhou, Henan, China; ^5^Department of Cardiology, Ankara Yildirim Beyazit, Ankara, Turkey; ^6^Department of Cardiology, California Pacific Medical Center, San Francisco, CA, USA; ^7^Interventional Electrophysiology, Scripps Clinic, La Jolla, CA, USA; ^8^Department of Cardiology, MetroHealth Medical Center, Case Western Reserve, University School of Medicine, Cleveland, OH, USA; ^9^Division of Cardiology, Stanford University, Stanford, CA, USA; ^10^Dell Medical School, University of Texas, Austin, TX, USA; ^11^Department of Biomedical Engineering, Cockrell School of Engineering, University of Texas, Austin, TX, USA; ^12^Division of Cardiology, Department of Medicine, University of Texas Health Sciences Center, San Antonio, TX, USA

**Keywords:** Catheter ablation, electrophysiological study, fluoroscopy, imaging

## Abstract

In the past decade, the use of interventional electrophysiological (EP) procedures for the diagnosis and treatment of cardiac arrhythmias has exponentially increased. These procedures usually require fluoroscopy to guide the advancement and frequent repositioning of intracardiac catheters, resulting in both the patient and the operator being subjected to a considerable degree of radiation exposure. Although shielding options such as lead gowns, glasses, and pull-down shields are useful for protecting the operator, they do not lessen the patient’s level of exposure. Furthermore, the prolonged use of lead gowns can exponentiate the onset of orthopedic problems among operators. Recent advancements in three-dimensional cardiac mapping systems and the use of radiation-free imaging technologies such as magnetic resonance imaging and intracardiac ultrasound allow operators to perform EP procedures with minimal or even no fluoroscopy. In this review, we sought to describe the state of fluoroless procedures in EP practice.

## Introduction

Interventional electrophysiological (EP) procedures are widely used for both the diagnosis and treatment of various types of cardiac arrhythmias. These procedures require the use of intracardiac catheters, which are typically advanced and frequently repositioned under fluoroscopy guidance (conventional approach). Procedural and, more notably, fluoroscopic times have progressively lengthened because of the increasing complexity of EP procedures requiring detailed mapping and/or extensive ablation therapy. Therefore, significant efforts have been made in the past few years to reduce radiation exposure among both patients and operators alike. For instance, the incorporation of advanced imaging modalities such as real-time ultrasonography (US), intracardiac echocardiography (ICE), and three-dimensional electroanatomic mapping (EAM) systems have greatly reduced the requirements for fluoroscopy in EP laboratories without any significant difference observed in the safety and efficacy of the procedures.^1–3^ Although all interventional EP centers should aim for fluoroscopy use to be as low as reasonably achievable (per the ALARA principle), variability in fluoroscopy times can be observed among centers as well as within the same center depending in part on procedure complexity and operator expertise.^4^ In a previous meta-analysis, Yang et al.^5^ demonstrated that there was no significant difference between minimal or zero fluoroscopy and conventional ablation with regard to procedural time, acute and long-term success rates, complications, and recurrence rates.

In order to implement minimal- or zero-fluoroscopy techniques in routine daily practice, physicians should be trained to use EAM systems and advanced intraoperative imaging modalities as early as possible—for example, either during their initial EP training or in supplementary hands-on courses. One of the drawbacks of this approach is the potential necessity of rescue fluoroscopy during emergent conditions while the operators and other laboratory staff are not wearing the appropriate lead protection.^6^ In the last decade, there has been a significant increase in the number of EP procedures performed with minimal or zero fluoroscopy.^1,7–9^ Any type of cardiac arrhythmia, including supraventricular tachycardias (SVTs), atrial fibrillation (AF), and ventricular arrhythmias (VAs), can in theory be ablated with a fluoroless approach in experienced centers. These fluoroless procedures are exceedingly significant in specific patient subpopulations who are at a higher risk of adverse effects from radiation exposure such as pregnant women, patients with obesity, and pediatric patients.^4,7,8,10–15^ In this review, we sought to describe how fluoroless procedures are carried out and highlight the new tools available to EP operators in this area of practice. In addition, we present the most recent data on fluoroless procedure outcomes.

## Supportive imaging tools during fluoroless procedures

Many operators are used to relying heavily on fluoroscopic guidance during catheter manipulations. This constitutes a significant barrier to the successful implementation of the fluoroless approach wherein alternative nonradiographic imaging tools are utilized instead of fluoroscopy. For instance, at the beginning of the procedure, real-time ultrasound can be used to obtain jugular or femoral venous access in order to reduce access site complications. Next, two-dimensional phased-array intracardiac echocardiography (CARTO Sound; Biosense-Webster Inc., Diamond Bar, CA, USA), three-dimensional ICE-based imaging, fast anatomical mapping (FAM) or EAM systems, and medical positioning systems (MediGuide, Inc., Wilmington, DE, USA) are used separately or in conjunction with one another as necessary based on the type of procedure and preference of the operator.^1,2,6–8,16–18^ Preprocedural segmented computerized tomography (CT) or magnetic resonance imaging (MRI) can be integrated with EAM systems to limit fluoroscopy use. In a limited number of cases, fluoroless catheter ablations guided by three-dimensional transesophageal echocardiography (TEE) have been reported, including for typical atrial flutter (AFL), AF, accessory pathway, and ischemic ventricular tachycardia (VT) ablation.^19,20^ These imaging tools are used to visualize the anatomy of the cardiac chambers of interest and, in the case of the EAM, can also be used to study the electrical activity within the cavity in order to better guide the ablation procedure. Visualization of the chambers’ anatomy is helpful to understand the exact location of the catheter with respect to the chamber wall. Diagnostic catheters can be appropriately positioned in standard locations simply with the help of ICE, CT/MRI, medical positioning system, or EAM system annotations made on anatomical images. Overall, these tools can increase operators’ confidence regarding the safety of the procedure.

It has been suggested that a fluoroless approach may prolong the duration of the procedure. Although this may be true during the initial learning phase, prospective comparisons do not support this notion.^17^ Finally, in some specific conditions such as when treating patients with pacemaker/implantable cardioverter-defibrillator leads, interatrial septal defect closure devices, complex anatomic variations, or poor resolution achieved with fluoroless imaging tools, minimal fluoroscopy may be required to safely complete the procedure.^2^ Therefore, these procedures may be categorized as minimal- or zero-fluoroscopy based on specific needs.

## State of fluoroless procedures for supraventricular tachyarrhythmias

Most of the fluoroless procedures reported in the literature represent single-center experiences involving patients with right-sided supraventricular tachyarrhythmias such as atrioventricular nodal reentrant tachycardia (AVNRT), accessory pathways, typical/atypical AFL, and right atrial tachycardia (AT)^1,21–43^ **(Table 1)**. Less frequently shared are reports of fluoroless ablations of AF **(Table 2)** left-sided atrial tachyarrhythmias, and VAs **(Table 3)**. Although many operators are now experienced in the use of ICE for cardiac ablations, there are technical difficulties in the visualization of left atrial or left ventricular structures from the standard right atrial location of the ICE catheter. This limits the routine use of ICE in guiding left-sided ablation procedures. There have been several papers published on the role of three-dimensional EAM and/or other imaging tools to reduce fluoroscopy use during SVT ablations. However, these usually involve the reduced or minimal use of fluoroscopy, with reports of zero-fluoroscopy procedures remaining scarce.^9^ Children and newborns are a special subpopulation with a higher lifelong cumulative risk of radiation-related morbidity given their longer life expectancy as compared with adults. In these very young patients, minimal or zero-fluoroscopy approaches, usually with the use of three-dimensional EAM and ICE, have been implemented earlier and more rapidly than in adult patients.^44^ This was possible because most of the cardiac arrhythmias observed in children are SVT with a right-side origin (> 90% of cases are AVNRT), which can be easily treated with fluoroless procedures. Herein, we discuss the current state of fluoroless approaches in SVTs including AVNRT, accessory pathways, AFL, and ATs among pediatric and adult populations.

Ruiz-Granell et al.^34^ was one of the first study groups to report on the use of an uncomplicated, zero-fluoroscopy approach involving EAM during atrioventricular node ablation and permanent pacemaker implantation. Shortly thereafter, Earley et al.^35^ showed that the involvement of three-dimensional EAM systems significantly reduced procedural and fluoroscopy times in a variety of conditions requiring ablation including AVNRT, AVRT, AFL, and VT, with similar resulting success and complications rates as compared with those associated with the conventional approach. Specifically, the median radiation exposure was four minutes (range: 0–50 minutes) in the EAM-guided strategy group and 13 minutes (range: 12–46 minutes) in the conventional strategy group.

Elsewhere, Gist et al.^36^ described their learning curve over time during the transition to fluoroless ablations of AVNRT. In their study, 62 consecutive patients who underwent fluoroless cryoablation of AVNRT between December 2005 and August 2008 were included. The first 12 months since technique introduction were considered the “early era” (December 2005–December 2006; n = 27), whereas the “recent era” included the following 20 months (January 2007–August 2008; n = 35). Although no significant procedural complications were reported regardless of the “era,” a significant reduction in procedural time was observed over time [early era: 202 (100–419) minutes versus recent era: 160 (78–332) minutes]. The recurrence rates were 15% in the early era and 8% in the recent era, respectively.

Data from a multicenter prospective catheter ablation registry including both children and adults were presented by Stec et al.,^37^ where 179 out of 188 procedures were performed without fluoroscopy. Among these procedures, an acute success rate of 98% and a long-term success rate of 93% were achieved without major complications; these results were similar to those in the fluoroscopy-guided control group. Further, Fernandez-Gomez et al.^38^ demonstrated that a fluoroless approach using the EnSite™ NavX™ system (Abbott Laboratories, Chicago, IL, USA) was feasible, safe, and effective in a total of 340 procedures, including 153 typical AFL, 146 AVNRT, 35 AVRT, four incisional AFL, and two focal AT cases, respectively, during a six-year period. The authors additionally reported a high acute success rate (99.1%) with zero fluoroscopy applied in 94.7% of the procedures and a mean procedural duration of 110.5 minutes ± 51.8 minutes.

Of note, a multicenter study has shown a higher success rate of three-dimensional EAM-guided procedures as compared with procedures directed by fluoroscopy alone (97% versus 91%) for the ablation of accessory pathways in a pediatric population, with no significant difference in recurrence (5% versus 9%) or complication rates (0.3% versus 0.4%).^39^ In another multicenter study including 442 consecutive adult patients with SVT (43% AVNRT; 35% right-sided AFL; 11% accessory pathway; and 11% AT, atypical AFL, or VT), Giaccardi et al.^40^ also demonstrated the efficacy (acute success rate of 96% versus 97%) and safety (complication rate of 4.4% versus 2.1%) of near-zero fluoroscopy with the aid of the EnSite™ Velocity™ system (Abbott Laboratories, Chicago, IL, USA) in comparison with the conventional approach, without a significant increase in procedural duration (91 ± 52 minutes versus 87 ± 57 minutes). Importantly, 14% of the patients required rescue fluoroscopy because of difficult venous access, need to confirm catheter stability and location, frequent coronary sinus (CS) catheter dislocation, problems with the EAM system, and a need to check the positioning of guidewires. In 2019, the same group reported their long-term outcomes with a fluoroless approach over six years of experience.^41^

In the Radiation Exposure Reduction in SVT Ablation (NO-PARTY) study,^42^ a total of 262 patients undergoing EP study for supraventricular tachycardia (no AF) were randomized to a minimal fluoroscopic approach with the EnSite™ NavX™ navigation system (Abbott Laboratories, Chicago, IL, USA) or to a conventional approach procedure. The study results clearly showed similar degrees of safety and efficacy for both approaches with no radiation exposure in the minimal fluoroscopic approach group.

After demonstrating the feasibility, safety, and efficacy of fluoroless catheter ablation in 60 patients,^43^ Razminia et al.^1^ presented their five-year fluoroless catheter ablation experience in 500 consecutive patients with various types of cardiac arrhythmias (n = 639 arrhythmias) including AVNRT (n = 31), AVRT (n = 79), macro-AT (n = 188), focal AT (n = 111), AF (n = 186), VT (n = 14), and ventricular premature contractions (VPCs) (n = 30). The procedures were performed primarily using ICE and three-dimensional EAM guidance. Although there were no major complications in the AVRT, AVNRT, VT, and macro-AT patients, major complication rates of 1.7% in focal AT, 1.6% in AF, and 3.3% in VPC patients, respectively, were reported. No death events were reported in any patient group. Success rates were also consistent with previously published findings within each cardiac arrhythmia type. The mean procedural duration was significantly reduced over the years from 209.6 minutes in 2011 to 114.2 minutes in 2016.

## State of fluoroless procedures for atrial fibrillation

**Figure 1:**
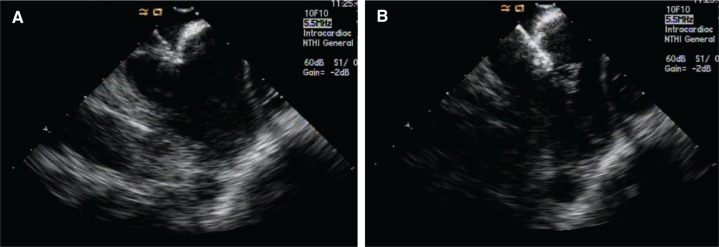
**A:** The transseptal needle tip is in the desired area (tenting) of the fossa ovalis on ICE. **B:** Radiofrequency energy is delivered to cross the septum.

The fluoroless approach is less common in AF ablation procedures in comparison with SVT ablations **(Tables 1 and 2)**. This may be in part due to the technical limitations of current imaging modalities to appropriately visualize left-sided structures as well as the greater complexity inherent in performing left-sided ablation procedures even when using the conventional approach.^2^ Moreover, operators are often more confident when performing certain maneuvers under fluoroscopic guidance such as the pull-back of transseptal sheaths in patients with intracardiac pacemaker/implantable cardioverter-defibrillator leads or transseptal puncturing in difficult anatomies. However, exposure to ionizing radiation in the fluoroscopic approach is higher in AF ablations as compared with in the ablation of other supraventricular tachycardias.^4^ Thus, the implementation of near-zero or zero fluoroscopy in AF ablation procedures would be critical to reduce the degree of radiation exposure for both patients and operators. Depending on the availability and operator’s experience with different noninvasive imaging tools such as two- and three-dimensional ICE, three-dimensional TEE, EAM, CT, or MRI segmentation and integration, ablations can be performed for the treatment of AF without using fluoroscopy. A simple two-dimensional ICE approach can assess intracardiac structures, guide a transseptal puncture, evaluate the distance between the catheter tip and the wall contact, and assess for procedural complications such as pericardial effusion. Operators mainly focus on the transseptal puncture as a critical and rate-limiting step in fluoroless AF ablations. However, there are alternative techniques available depending upon the experiences of the operators that are able to reduce fluoroscopy during transseptal puncture. In summary, these include, after positioning the ICE catheter in the right atrium, obtaining sound contours from the aortic root, the ostium of the CS, and the fossa ovalis; limited FAM of the CS, superior vena cava (SVC), and interatrial septum; positioning of the CS catheter; placement of the long-wire in the SVC per the guidance of ICE; advancement of the transseptal sheath and dilator over this wire by observing them in the SVC using ICE images; conducting advancement of a blunt transseptal needle (Baylis Medical, Toronto, Ontario, Canada) via a transseptal sheath; visualizing the uncovered needle tip on the mapping system; and performing withdrawal of the needle and sheath until the needle tip is in the desired area of the fossa ovalis on ICE and RF energy can be delivered to cross the septum **(Figure 1)**.^45^

In 2013, the Leipzig group reported their experience using MediGuide™ technology as a nonfluoroscopic imaging tool (MediGuide, Inc., Wilmington, DE, USA) for AF ablation procedures in 80 patients. The total procedural time was 167 minutes ± 47 minutes, with a median of 4.6 minutes of fluoroscopy to complete background loops, transseptal puncture, confirmation of the transseptal sheath position, and manipulation of the circular mapping catheter. Ultimately, in this patient cohort, there were only three (4%) minor complications.^46^ Elsewhere, Sommer et al.^47^ reported their large-volume experience with MediGuide™ four-dimensional navigation technology (MediGuide, Inc., Wilmington, DE, USA) including 1,000 patients who underwent AF ablation between 2012 and 2017, where the median fluoroscopy time was 0.9 minutes ± 2.7 minutes, with an acceptable complication rate of 2%.^48^ Completely fluoroless procedures were performed by Razminia et al.^1^ on 500 patients with supraventricular tachycardia, AF, premature ventricular complexes, and VT. Thanks to the supportive role of ICE, EAM, and intracardiac electrograms, an acceptable success rate was achieved with similar procedural duration times as compared with those of the conventional approach. The risk of complications also remained unchanged.

Small case series evaluating fluoroless approaches for the ablation of paroxysmal AF have also been reported.^17,49–52^ In 245 patients with paroxysmal AF, Lyan et al.^50^ reported that the fluoroless approach had similar procedural times as compared with fluoroscopy-guided ablations (108.8 ± 18.2 minutes in the fluoroless group versus 113.6 ± 26.8 minutes in the fluoroscopy-guided group). Three of 245 patients (1.2%) in the fluoroless group developed cardiac tamponade and required rescue fluoroscopy during pericardiocentesis.

In a retrospective analysis of five years of experience including 186 AF patients (including 150 treated with radiofrequency and 36 treated with cryoballoon), Razminia et al.^1^ reported the achievement of a total of 194 minutes of procedural time, a 1.6% rate of major complications (two cases of cardiac tamponade and one case of atrioesophageal fistula), and a 22.6% rate of recurrence.

Different from other patient series, O’Brien et al.^53^ reported their experience with a fluoroless approach using three-dimensional EAM and TEE rather than ICE in a total of 55 AF patients. The total procedural time was similar to that in the fluoroscopic approach group used as a control population and was consistent with previous series performed using ICE instead of TEE. The complication rate was also acceptable.

Liu et al.^54^ performed AF ablation in a total of 200 consecutive patients (AFL and nonpulmonary-vein triggers in 82% and paroxysmal AF in 55%) by using ICE, a non-navigation circular catheter, and contact force–sensing ablation catheters without anatomic mapping. The mean procedural time was 106.2 minutes ± 23.2 minutes, with a success rate of 76% at a median follow-up of 11 months. The complication rate was low (1% for minor complications) and the novel approach was cost-saving (ICE and circular mapping catheters were reprocessed). Furthermore, there were no adverse events related to the intracardiac leads in 19 of 200 patients with intracardiac devices.

Sadek et al.^55^ more recently published their experience with a fluoroless approach in a relatively complex patient population including 70 complex left AT (33 with persistent AF and six with left AFL) and 10 VT (60% with scar-mediated VT) ablations performed using ICE and EAM guidance, which had a 100% acute success rate, similar procedural times as compared with when using fluoroscopic guidance, and no complications. Despite such a limitation, the authors added to the evidence regarding the feasibility of a fluoroless approach in complex cardiac arrhythmias, including a more complex atrial substrate and a higher burden of atrial scar, characteristics which require more extensive ablation as compared with standard pulmonary vein isolation.

## State of fluoroless procedures for ventricular arrhythmias

Although the fluoroless approach has been frequently deployed in both children and adults with SVTs, its use in patients with VAs has so far been limited **(Table 3)**. This can be attributed to various reasons including limitations of supportive imaging tools for the study of ventricular anatomy, complexity of ventricular anatomy, limited experience of the operators, presence of intracardiac devices with the risk of lead dislocation, and difficulty in left ventricular access via both retrograde or anterograde approaches. Nevertheless, the use of a fluoroless approach for the ablation of VAs is destined to grow due to the increasing number of patients requiring ventricular ablations and the improvement of advanced imaging technologies for the visualization of ventricular components and neighboring structures. Similar to the procedure for SVT, the fluoroless approach in patients with VA is based on advanced imaging tools like ICE, three-dimensional EAM, and the integration of CT or MRI segmentation such as the CartoUnivu™ Module (Biosense Webster, Diamond Bar, CA, USA).^56^ With implementation of these imaging tools, fluoroless procedures have been successfully performed in idiopathic VT case series,^57^ with similar procedural efficacy rates and durations as compared with those achieved using a fluoroscopic approach. Notably, 5% to 6% of patients initially treated with the fluoroless approach still required rescue fluoroscopy.

As mentioned in a previous section, Sanchez et al.^31^ reported their single-center experience with a fluoroless approach using three-dimensional EAM in all patients and ICE in 70.4% of patients. The study included a total of 10 ablation procedures for VAs including VPCs and VTs with a mean procedure time of 150 minutes ± 45 minutes and no complications. Furthermore, Sadek et al.^55^ reported a total of 10 fluoroless VT ablations (60% involving scar-mediated VTs and 1% being ARVC-mediated) using ICE and three-dimensional EAM. All patients with outflow-tract VPCs demonstrated no recurrence during follow-up, and the treatment success rate was 83% in patients with scar-mediated VTs. As most of these patients have intracardiac devices, operators should be careful not to dislodge the leads during catheter insertion or during transseptal access. The leads should be carefully visualized before and just after the ablation procedure to confirm the positioning of the leads.

## State of fluoroless procedures for special populations

As previously mentioned, fluoroless catheter ablation procedures are particularly important when it comes to specific patient subgroups such as children, pregnant women **(Table 4)**, or patients with obesity. In pregnant women, the avoidance of radiation exposure is especially critical during the first trimester of pregnancy because of the higher risk for fetal adverse effects. Therefore, the risks and benefits of catheter ablation procedures in these subpopulations should be carefully evaluated by the treating physicians. If catheter ablation is planned, operators should strongly consider using a fluoroless approach.

Although there are several single-center or multicenter studies available that describe the feasibility, safety, and efficacy of fluoroless procedures in children, data on pregnant women are available from just a few case reports and case series. Nevertheless, data in pregnant women diagnosed with SVTs such as AVNRT, AVRT, PJRT, AT, AF, or ventricular arrhythmias including VPCs and/or VTs seem to support the performance of fluoroless three-dimensional EAM- or ICE-guided procedures in between 10 weeks and 38 weeks of pregnancy without any associated complications for the patient or the baby. Procedural times were consistent with previous reports on nonpregnant patients undergoing the same procedure.^58–68^ The ablation of ventricular arrhythmias in two pregnant patients, one with idiopathic right ventricular outflow-tract (RVOT) VPC and the other with VT (RVOT anterior) due to electrical storm in ARVC, were also successfully performed with the use of three-dimensional EAM.

## State of reduced fluoroscopy during cardiac implantable electronic device placement

Imaging of the venous puncture site, wire, and lead placement location is necessary during cardiac implantable electronic device (CIED) implantations, and fluoroscopy remains by far the most commonly used approach, particularly for the insertion of cardiac resynchronization therapy devices. By involving ultrasonographic guidance during transvenous access and the placement of wires and sheaths as well as making simple modifications to modern X-ray system settings by way of further advancements in technology, the total radiation dose can be reduced significantly.^69–71^ As image quality demands are usually modest for most CIED implantations, the use of an ultralow frame rate (2–4 frames/s) and antiscatter gridless (removal of antiscatter grid setting) radiation protocols significantly reduce the radiation dose needed during the implantation of CIEDs without an increase in procedural duration or complication rates.^71^

## Conclusions

“Near-zero” or “zero” fluoroscopic procedures that implement advanced imaging tools, including three-dimensional EAM and ICE, have been shown to be as safe and effective as the traditional fluoroscopy-guided approach. Fluoroless procedures not only avoid the unnecessary exposure of patients and health care providers to radiation but also limit the prolonged use of lead gowns, which results in a reduced incidence of musculoskeletal complications among operators. With adequate training, this approach can be safely and effectively implemented without any increase in procedural time. Nevertheless, “rescue” fluoroscopy should readily be made available at all times in the case of complications such as cardiac tamponade, difficulties with advancing or localizing catheters, or technical problems with the EAM systems or other imaging modalities. The fluoroless approach should be decidedly favored in selected subgroups including children, patients with obesity, and pregnant women in whom the effects of radiation exposure can be especially adverse and can be applied in the treatment of a variety of arrhythmia types **(Table 5)**. Large studies will be necessary to further validate the fluoroless approach, particularly for the purpose of VT ablation.

## Figures and Tables

**Table 1:** Studies Including SVT Patients in Whom Near-zero- or Zero-fluoroscopy Catheter Ablation Was Performed

Study	No. of Fluoroless Patient(s)	Arrhythmia Type(s)	Imaging Modality	Procedural Time	Fluoroscopy Time for Study Group	Follow-up Success Rate(s)	Complication Rate(s)
Scaglione et al.^7^	44	Concealed accessory pathway	EAM	94.4 ± 4.4 min	100% zero fluoroscopy	84.1% at 16.0 ± 11.7 months	0%
Walsh et al.^9^	50	AVNRT, AFL, accessory pathway, AT, AV-node ablation	EAM	57 min	100% zero fluoroscopy	98% at 4.9 months	0%
Ozturk et al.^11^	9	Mahaim pathways	EAM	249 ± 90 min	4.5 min, 66.7% zero fluoroscopy	77.8% at 25 months	0%
Elkiran et al.^12^	39	AT	EAM, fluoroscopy	184.23 ± 60.19 min	5.53 ± 5.22 min, 64.1% zero fluoroscopy	87.2% at 51.35 ± 12.62 months	2.5%
Drago et al.^22^	21	Right-sided accessory pathway/WPW	EAM, fluoroscopy	NA	9.2 ± 7.7 min in 12 patients, 42.8% zero fluoroscopy	100% at 15 ± 7 months	0%
Smith et al.^23^	30	AVNRT, AVRT	EAM, fluoroscopy	3.27 hours	1.05 ± 2.9 min, 80% zero fluoroscopy	87% at 3 months	Transient AV block in 30%
Clark et al.^24^	10	Left-sided accessory pathway	EAM, TEE, fluoroscopy	266 min	100% zero fluoroscopy	10% at 6 months	0%
Scaglione et al.^25^	21	AVNRT	EAM, fluoroscopy	70 ± 20 min	45 s and 50 s, 90.4% zero fluoroscopy	76.2% at 25 months	0%
Bigelow et al.^26^	2 neonates	WPW	EAM	112 min and 110 min	100% zero fluoroscopy	100% at 14 months and 7 months	0%
Alvarez et al.^27^	50	AVNRT	EAM, fluoroscopy	152 ± 35 min	98% zero fluoroscopy	96% in EAM group at 6 months	2%
Alvarez et al.^28^	83	Typical AFL	EAM, fluoroscopy	141 ± 47 min	90.4% zero fluoroscopy	96.25% at 18 ± 12 months	6%
Sommer et al.^29^	24	AVNRT, AVRT, WPW, AT, Typical AFL	EAM, MediGuide, fluoroscopy	70 ± 25 min	0.5 ± 1.5 min	100% (acute)	0%
Jan et al.^30^	43	AVNRT, AT, AVRT, accessory pathway	EAM, ICE, fluoroscopy	126 ± 53 min	100% zero fluoroscopy	90.3% at 10 ± 3 months	0%
Ruiz-Granell et al.^34^	1	AV-node ablation + pacemaker implantation	EAM	87 min	< 1 min	100%	0%
Gist et al.^36^	62	AVNRT	EAM	202 ± 76 min in early vs. 160 ± 61 min in recent era		88.7% at 12 months	Transient AV block in 44% in early vs. 38% in recent era
Stec et al.^37^	188	AVNRT, AVRT, WPW, AFL, AT, double tachycardia	EAM, fluoroscopy	63 ± 26 min	95.2% zero fluoroscopy	93.5% at 8.0 ± 5.2 months	0%
Fernandez-Gomez et al.^38^	328	AVNRT, AVRT, Typical AFL, incisional AFL, AT	EAM, fluoroscopy	110.5 ± 51.8 min	94.7% zero fluoroscopy	96.5% at 6 months	0.3%
Ceresnak et al.^39^	651	Accessory pathway, WPW	EAM, fluoroscopy	NA	100% zero fluoroscopy	95% at 1.0 ± 0.9 years	0.3%
Giaccardi et al.^40^	297	AVNRT, AFL, accessory pathway	EAM, fluoroscopy	87 ± 57 min	14 s ± 6 s, 86% zero fluoroscopy	98% (acute)	2.1%
Casella et al.^42^	134	AVNRT, accessory pathway, AFL, AT	EAM, fluoroscopy	NA	0–12 s, 72% zero fluoroscopy	97% at 12 ± 4 months	0%

AF: atrial fibrillation, AFL: atrial flutter, AT: atrial tachycardia, AV: atrioventricular, AVNRT: atrioventricular nodal reentrant tachycardia, AVRT: atrioventricular reentrant tachycardia, EAM: electroanatomic mapping, ICE: intracardiac echocardiography, NA: not applicable, PJRT: permanent junctional reciprocating tachycardia, VPC: ventricular premature contraction, WPW: Wolf–Parkinson–White syndrome.

**Table 2:** Studies Including AF Patients in Whom Near-zero- or Zero-fluoroscopy Catheter Ablation Was Performed

Study	No. of Fluoroless Patient(s)	Arrhythmia Type(s)	Imaging Modality	Procedural Time	Fluoroscopy Time for Study Group	Follow-up Success Rate(s)	Complication Rate(s)
Percell et al.^9^	72	AF	EAM, ICE	210.38 min	0.1 min	68% at 3 months	5%
Bulava et al.^17^	40	AF	EAM, ICE	92.5 ± 22.9 min	8 s, 97.5% zero fluoroscopy	85% at 12 months	0%
Yamada et al.^18^	15	AF	EAM, CT, MediGuide™	NA	NA	82% at 12.2 ± 4.5 months	0%
Rolf et al.^46^	80	AF	EAM, MediGuide™, fluoroscopy	167 ± 47 min	4.6 min	NA	4% minor
Sommer et al.^47^	1000	AF	EAM, MediGuide™, fluoroscopy	120 min	0.9 min	NA	2%
Reddy et al.^49^	20	AF	EAM, ICE, fluoroscopy	244 ± 75 min	244 ± 75 min	90% at 6.1 ± 2.2 months	0%
Lyan et al.^50^	245	AF	EAM, ICE, fluoroscopy	108.8 ± 18.2 min	35, 44, and 107 s, 98.8% zero fluoroscopy	73.5% at 15.2 ± 4.1 months	1.2% cardiac tamponade in the no-fluorocopy group
Ferguson et al.^51^	22	AF	EAM, ICE, fluoroscopy	208 min	2–16 min, 86.3% zero fluoroscopy	76% at 7 months	0%
O’Brien et al.^53^	69	AF	EAM, TEE, fluoroscopy	124.04 ± 45.41 min	54% (development phase) vs. 93.3% (implementation phase) zero fluoroscopy	NA	4.3%
Liu et al.^54^	200	AF	EAM, ICE	106.2 ± 23.2 min	100% zero fluoroscopy	76% at 11 months	1%

AF: atrial fibrillation, CT: computed tomography, EAM: electroanatomic mapping, ICE: intracardiac echocardiography, NA: not applicable, TEE: transesophageal echocardiography.

**Table 3:** Studies Including VA Patients in Whom Near-zero- or Zero-fluoroscopy Catheter Ablation Was Performed

Study	No. of Fluoroless Patient(s)	Arrhythmia Type(s)	Imaging Modality	Procedural Time	Fluoroscopy Time for Study Group	Follow-up Success Rate(s)	Complication Rate(s)
Akdeniz et al.^13^	35	VAs	EAM, fluoroscopy	175 ± 67 min	2.35 ± 1.89 min, 54.3% zero fluoroscopy	80% at 15.9 ± 7.1 months	8.6%
Ozyilmaz et al.^14^	17	VTs	EAM	169.3 ± 43.2 min	8.6 ± 10.8 min, 35.3% zero fluoroscopy	82.4% at 8.5 ± 7.6 months	5.9%
Cano et al.^56^	41	VT (endo- and/or epicardial)	EAM, CARTO-UNIVU Module, fluoroscopy	193 ± 62 min for ischemic VT, 217 ± 59 min for nonischemic VT	6.08 min, 32% zero fluoroscopy	92.7% at 217 days	4.9%
Wang et al.^57^	163	VAs	EAM	77.1 ± 33.8 min	94.4% zero fluoroscopy	88.1% at 5.4 ± 3.9 months	0.6% major

EAM: electroanatomic mapping, VA: ventricular arrhythmia, VT: ventricular tachycardia.

**Table 4:** Procedural and Follow-up Details of Pregnant Women with Different Types of Arrhythmias in Whom Near-zero- or Zero-fluoroscopy Catheter Ablation Was Performed

Study	No. of Fluoroless Patient(s)	Arrhythmia Type(s)	Imaging Modality	Procedural Time	Fluoroscopy Time for Study Group	Follow-up Success Rate(s)	Complication Rate(s)
Bongiorni et al.^58^	1 pregnant (10^th^ week)	AVNRT	ICE	80 min	100% zero fluoroscopy	NA	0%
Szumowski et al.^59^	9 pregnant (12^th^–38^th^ week)	PJRT, AT, AVNRT, WPW	EAM, fluoroscopy	56 ± 18 min	42 ± 37 s, 33.3% zero fluoroscopy	100% at 43 ± 23 months	0%
Ferguson et al.^60^	1 pregnant (27^th^ week)	AT	EAM, ICE	NA	100% zero fluoroscopy	100% at 4 weeks	0%
Manjaly et al.^61^	1 pregnant (15^th^ week)	VF (preexcited AF) and AVRT	EAM	NA	100% zero fluoroscopy	100% (acute)	0%
Leiria et al.^62^	1 pregnant (26^th^ week)	AVRT	EAM	140 min	100% zero fluoroscopy	100% (acute)	0%
Zuberi et al.^63^	1 pregnant (30^th^ week)	AT	EAM	NA	100% zero fluoroscopy	100% at 3 years	0%
Chen et al.^66^	2 pregnant (25^th^ and 31^st^ weeks)	VPCs, AVRT	EAM	41 min and 71 min	100% zero fluoroscopy	100% at 12 months	0%
Stec et al.^67^	1 pregnant (23^rd^ week)	ARVC, VT	EAM, fluoroscopy	NA	90 s	100% at 12 months	0%
Omaygenc et al.^64^	3 pregnant (12^th^, 21^st^, and 30^th^ weeks)	AVRT, AVNRT	EAM	29, 36, and 45 min	100% zero fluoroscopy	100% (acute)	0%
Bigelow et al.^65^	1 pregnant (22^nd^ week)	AVNRT	EAM	186 min	100% zero fluoroscopy	100% at 26 months	0%
Kozluk et al.^68^	11 pregnants (16^th^–32^nd^ weeks)	AVNRT, WPW, PJRT, AT, VPCs	EAM	NA	100% zero fluoroscopy	100% (acute)	0%

AF: atrial fibrillation, ARVC: arrhythmogenic right ventricular cardiomyopathy, AT: atrial tachycardia, AVNRT: atrioventricular nodal reentrant tachycardia, AVRT: atrioventricular reentrant tachycardia, EAM: electroanatomic mapping, ICE: intracardiac echocardiography, NA: not applicable, PJRT: permanent junctional reciprocating tachycardia, VPC: ventricular premature contraction, WPW: Wolf–Parkinson–White syndrome.

**Table 5:** Studies Including SVT, AF, and VA Patients in Whom Near-zero- or Zero-fluoroscopy Catheter Ablation Was Performed

Study	No. of Fluoroless Patient(s)	Arrhythmia Type(s)	Imaging Modality	Procedural Time	Fluoroscopy Time for Study Group	Follow-up Success Rate(s)	Complication Rate(s)
Razminia et al.^1^	500	AF, AVNRT, AVRT, AT, VAs	EAM, ICE	194.4 min for AF	0.3 min, 99.5% zero fluoroscopy	77.3% for AF at 21.2 months; 78.6% for VT at 16.6 months	1% major, 0.6% minor
Tuzcu et al.^8^	183	AVNRT, AVRT, AT, Junctional tachycardia, VT	EAM, fluoroscopy	202.8 ± 83.1 min	63% zero fluoroscopy	90% at 44.9 ± 23.3 months	0.7%
Koca et al.^15^	78	AVNRT, accessory pathway, AT, VAs	EAM, fluoroscopy	153.1 ± 44.3 min	5.4 ± 3.15 min, 81.6% zero fluoroscopy	94.9% at 43.4 weeks ± 23.3 weeks	2.5%
Sanchez et al.^31^	107	SVT, AF, AFL, VAs	EAM, ICE	26 ± 50 min	100% zero fluoroscopy	NA	0%
Kozluk et al.^32^	45	AVNRT, WPW, AT, VAs	EAM, fluoroscopy	110 ± 43 min	84% zero fluoroscopy	89.5%	2.2% minor
Kipp et al.^33^	124	AVNRT, AVRT, AT, AFL, accessory pathways, VAs	EAM, fluoroscopy	NA	1.21 ± 1.18 min, 76% zero fluoroscopy	91.9% overall	3.81% minor
Earley et al.^35^	94	AVNRT, AVRT, AFL, Other (AT, VT)	EAM, fluoroscopy	90 min	6 min in CARTO and 4 min in NavX groups	86% in CARTO and 87% in NavX groups at 6^th^ week	2% in CARTO and 2% in NavX groups
Giaccardi et al.^41^	266	AVNRT, AVRT, accessory pathway, AFL, AT, VAs, AV-node ablation, AF	EAM	75 ± 0 min to 145 ± 15 min for different arrhythmias	100% zero fluoroscopy	90.8% at 2.9 ± 1.6 years	0.8%
Razminia et al.^43^	60	AF, AVNRT, AVRT, AFL, AT, VAs	EAM, ICE, fluoroscopy	NA	100% zero fluoroscopy	NA	1.6% minor
Haegeli et al.^52^	34	AF, VAs, AVNRT, Typical AFL	EAM, ICE, fluoroscopy	130 ± 50 min for AF	54% for AF and 62% overall zero fluoroscopy	NA	2.9%
Sadek et al.^55^	80	AF, AFL, VAs	EAM, ICE	225 ± 32 min	100% zero fluoroscopy	91.2% at 18.1 ± 3.6 months	0%
Wannagat et al.^72^	57	AVNRT, AVRT, AT, AFL, VAs	EAM	88.8 ± 31.4 min in no-TSP group, 131 ± 25.2 min in TSP group	1.6 ± 4.2 min in no-TSP group, 3.2 ± 1.5 min in TSP group	95.5% at 3 months in no-TSP group and 92.3% at 3 months in TSP group	0%

AF: atrial fibrillation, AFL: atrial flutter, AT: atrial tachycardia, AV: atrioventricular, AVNRT: atrioventricular nodal reentrant tachycardia, AVRT: atrioventricular reentrant tachycardia, EAM: electroanatomic mapping, ICE: intracardiac echocardiography, NA: not applicable, PJRT: permanent junctional reciprocating tachycardia, TSP: transseptal puncture, VA: ventricular arrhythmia, VT: ventricular tachycardia, VPC: ventricular premature contraction, WPW: Wolf–Parkinson–White syndrome.
